# Chronic pain: A big challenge

**DOI:** 10.1590/1516-3180.2024.1421.131223

**Published:** 2024-02-19

**Authors:** Telma Zakka, Hélio Papler, Paulo Manuel Pêgo-Fernandes

**Affiliations:** I MD, PhD in Sciences, Department of Neurology, University of São Paulo; Expert in chronic pain management as certified by the Brazilian Medical Association, São Paulo, São Paulo, Brazil; Coordinator of the Chronic Pelvic Pain Outpatient Clinic, Interdisciplinary Pain Center, Neurology Clinic, Hospital das Clínicas, School of Medicine, University of São Paulo, São Paulo, São Paulo, Brazil; President of the Pain Committee, São Paulo Medical Association, São Paulo, São Paulo, Brazil.; Department of Neurology, University of São Paulo, São Paulo, São Paulo, Brazil; Brazilian Medical Association, São Paulo, São Paulo, Brazil; Hospital das Clínicas, School of Medicine, University of São Paulo, São Paulo, São Paulo, Brazil; II MD, PhD, Senior Professor, Department of Surgery, Federal University of São Paulo (UNIFESP) São Paulo, São Paulo, Brazil.; III MD, PhD, Vice-director, School of Medicine, University of São Paulo, São Paulo, São Paulo, Brazil; Full Professor, Department of Cardiopneumology, School of Medicine, University of São Paulo, São Paulo, São Paulo, Brazil; Director of the Scientific Department, São Paulo Medical Association, São Paulo, São Paulo, Brazil.

Diagnosing and treating chronic pain is a major clinical challenge for healthcare professionals. Chronic pain affects patient recovery and functionality, resulting in dissatisfaction and frustration among patients and their providers.^1^ The major goals in treating chronic pain are to provide long-lasting relief, markedly reduce suffering, and improve functionality and health-related quality of life. The treatment should also minimize side effects and adverse events to provide more cost-effective care.^2^

Continuous pain is a multidimensional disorder that involves physical, cognitive, psychological, and behavioral aspects. Given its complex nature and treatment objectives, healthcare professionals are aware that any single or combined treatment is not curative.^2^

The treatment of chronic pain often involves different combinations of medications, physical rehabilitation, lifestyle changes, psychotherapy, advanced pain interventions, surgery, and complementary medicine. Multimodal therapy represents the evolution of care for patients with partial or incomplete responses to conventional treatment, occurring in a formal environment, such as in structured rehabilitation programs.^2^

Single-drug therapy for continuous chronic pain seldom provides satisfactory relief. Therefore, combined pharmacological treatment is important in multimodal pain management. Finding a balance between effective treatment and acceptable side effects is a key factor in the pharmacological treatment of pain.^3^

## EFFECTIVE TREATMENT FOR CHRONIC PAIN

An effective treatment is hard to define and rarely means complete pain remission.^2^ ^,^ ^3^ A review of a series of clinical trials on chronic pain sponsored by the pharmaceutical industry considered 30% pain reduction as clinically significant because patients reported a “much better” pain experience at this level.^2^ ^,^ ^3^

Many pharmacological agents used in chronic pain treatment are central nervous system depressants and can affect patients’ energy levels and impair mobility, memory, and ability to perform physical exercise, which are crucial factors for successful rehabilitation.^2^ ^,^ ^3^ Mechanisms underlying chronic pain greatly vary and are still poorly understood; however, they are often characterized by increased nervous system sensitivity and hyperexcitability, which can be treated with anticonvulsants, antidepressants, and opioids.

## MULTIMODAL ANALGESIA

Reducing the doses of pharmacological agents to decrease sedation is not always convenient for also reducing its analgesic effects. The inclusion of a second agent with an additive (synergistic) analgesic effect but no cumulative side effect profile allows physicians to prescribe lower doses without losing analgesic efficacy or increasing side effects.^4^ The use of combination pharmacotherapy (multimodal analgesia) has a broad evidence base in acute pain management that can be extended to the treatment of chronic pain.^4^

Combination therapy is an important and common approach in chronic pain treatment, and future improvements should include the development of clinical strategies to predict positive outcomes and optimization of combined and individualized therapy protocols. A prudent and rational approach before more information becomes available should include careful treatment individualization, potential risk monitoring, and patient follow-up. It is also worth bearing in mind that “all chronic pain was once acute,” and hence, it is important to control it.
